# Designing an Effective and Scalable UV-Protective Cooling Textile with Nanoporous Fibers

**DOI:** 10.1021/acs.nanolett.3c03055

**Published:** 2023-11-06

**Authors:** Kyuin Park, Margaret W. Frey

**Affiliations:** Department of Human Centered Design, College of Human Ecology, Cornell University, Ithaca, New York 14850, United States

**Keywords:** nanoporous fiber, UV protection, hierarchical structure, scalable cooling textile

## Abstract

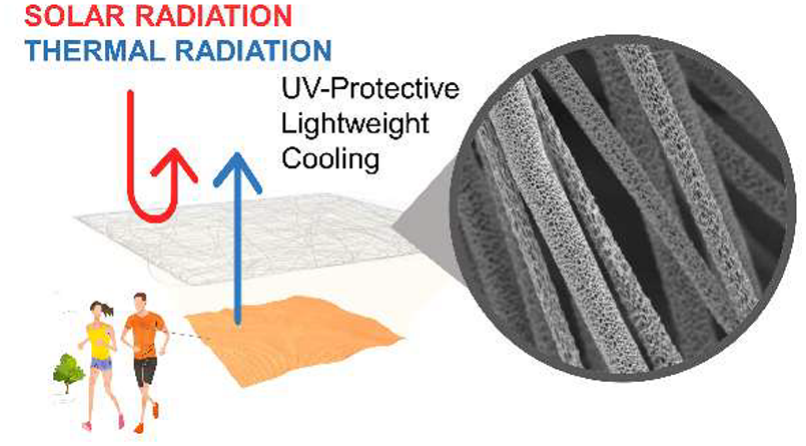

Although radiative cooling concepts guarantee reduction of air conditioning energy consumption by maximizing the scattering of solar radiation and dissipation of thermal radiation of a human body or building, large-scale implementation is challenging due to the need of radical adaptation in manufacturing processes, materials, and design. Here, we introduce an extremely thin layer of nanoporous microfibers without any additional materials or post-treatments. The optical and thermal effectiveness of porous fibers are presented to report a nondisruptive method of preventing the transmission of energy-intensive radiation such as ultraviolet radiation (UV) through textiles. Results show ∼1.4 °C cooling by adding 1 g/m^2^ (GSM) of porous fibers on a 160 GSM cotton t-shirt, and 91% of UV was prevented with 7.5 GSM of a porous fiber mat. This minimalistic additive approach would widen the scope of optical and radiative cooling research and accelerate both functional and sustainable materials research to be more accessible.

Ongoing global efforts to mitigate climate change focus on reducing heat-trapping greenhouse gas (GHG) emissions and aim for zero emission. Heat is not only a danger to health and safety but also triggers excessive consumption of energy toward air conditioning.^1−4^ GHG emission and use of air conditioning are common toxic cycles seen in larger cities, often called heat islands, where there are more surfaces being heated by solar radiation. The UN reported that energy used only for air conditioning accounts for 20% of the total energy used to operate buildings, and the operation of buildings accounts for 36% of the global electricity consumption and 37% of the global CO_2_ emission.^5^ Since human activities are not constrained to either indoors or outdoors but a combination of both while experiencing constant changes in surroundings and personal conditions, optimization of personal thermal comfort and safety is not simple.^6^

Passive radiative cooling is an electricity-free approach to cool buildings, objects, and human bodies. In recent decades, researchers have engineered materials and structures to make the most out of the thermodynamics of radiation for cooling indoors and outdoors during daytime and nighttime.^7,8^ One of the challenges radiative cooling technologies face in order to scale-up is the cost, which has correlation to the cost of raw materials, the availability of materials, the technique required to adapt in manufacturing processes, and, ultimately, the waste management.^8,9^ With the urgency for rapid impact, simplicity and scalability were emphasized in many studies by simplifying materials and processes and highlighting the structural modifications.^10−18^ For a personal thermally comfortable textiles, heat transfer model analysis has shown that efficient dissipation of long-wavelength infrared (LWIR, λ = 8–13 μm) is essential. Under the sun, high solar reflectivity (λ < 2.5 μm) becomes a dominating factor for thermal comfort.^13,19−22^ Peng et al.^21^ achieved 2.3 °C cooling with nanoporous polyethylene fibers, and Kim et al.^13^ demonstrated 12 °C cooling with submicron fibers with high solar reflectivity and thermal transparency for personal thermal comfort. Despite the high performance, another challenge that can be seen from scaling radiative cooling textiles is the undeniable gap between research and day-to-day clothing, which may be a burden for the manufacturer and an end user.

Fortunately, success stories in mass production of unconventional^23^ fiber material or dimensions, such as LifeLabs Design’s use of polyethylene^15,21,24^ and Teijin Frontier’s use of submicron fibers^25,26^ for thermal comfort, prove a positive outlook. In addition, concerns around the capability and mechanical durability of nanofibers have been addressed to validate the manufacturability and usability.^27−32^

Ultraviolet radiation (UV) is classified as carcinogenic to humans, causing immune suppression and skin cancers such as basal cell carcinoma, squamous cell carcinoma, and melanoma.^33−35^ While the prevention of heat may be considered a primary concern, in terms of energy conservation and thermal comfort, protection from UV is often overlooked. UV exposure is a threat to many polymers and construction materials, causing discoloration or degradation of the mechanical integrity.^36^ In efforts to address it, TiO_2_ and Al_2_O_3_ have been incorporated.^37−41^ However, the expansion of nanoparticle production and distribution gave rise to concerns around potential health hazards and contamination in the aquatic ecosystem.^42−44^

Here, we report on an extremely thin and light nanoporous microfiber layer to answer what structural design would exhibit passive cooling properties that address current challenges: requiring minimal use of material and avoiding disruptive/radical changes in processes. A hierarchical structure was created within fibers that maximizes the Mie resonant scattering. Aside from simulations,^15,18,19^ this is a novel experimental achievement that proves the effectiveness in scattering with minimal use of material. To quantify the accuracy, our fabrication method focused on (1) nanoscale pore creation on microfibers and (2) consistency and stability of fabricated fiber mats for feasible spectroscopic and thermal analyses. Without restricting to one specific material, or requiring any additional materials or any highly technical processes, this study aims to impact the current challenges of cost, manufacturability, and wearability by demonstrating the optical and thermal effectiveness of a minimal amount of porous fibers (0–10 GSM, grams/meter^2^) as potential additive fibers to be spun or knitted with conventional textiles.

Figure 1 illustrates the concept and fabrication method of an effective and scalable UV-protective cooling textile via nanoporous fibers electrospun from a single polymer source with no additional nanomaterials or other postprocessing methods. Figure 1a shows an expanded illustration of a thin layer of a nanoporous fiber mat on outdoor sportswear, where highly energy-intensive wavelengths in the solar spectrum are scattered and the natural thermal radiation from underneath transmits through freely for effective human body cooling.^7,13,19,21^Figure 1b shows a schematic of an electrospinning setup to fabricate both nonporous fiber (NPF) and porous fiber (PF) mats. The polymer used in this study is biobased and biodegradable polylactic acid (PLA).^45,46^ It is one of the most widely used biobased polymers with well-established waste management systems.^46^ Based on Mie scattering theory, the scattering efficiency is highest when *d* ≈ λ, where *d* is the size of feature and λ is the wavelength.^23,47^ Along the same line, reflection at λ can be minimized by controlling *d* to be much smaller than λ for the radiation at λ to experience weak Rayleigh scattering that, in comparison to strong Mie scattering, is negligible.^19,47^ Therefore, the proposed additive-fiber approach to a conventional manufacturing process would be suitable for any existing or developing materials and would exhibit comparable solar reflective properties with similar nanoscale and microscale features.^13,18,21^ Material with weak absorbance and high transmittance in an LWIR spectrum, such as polyethylene, would dissipate thermal radiation through the atmospheric window more efficiently.^15,19,21,23^

**Figure 1:**
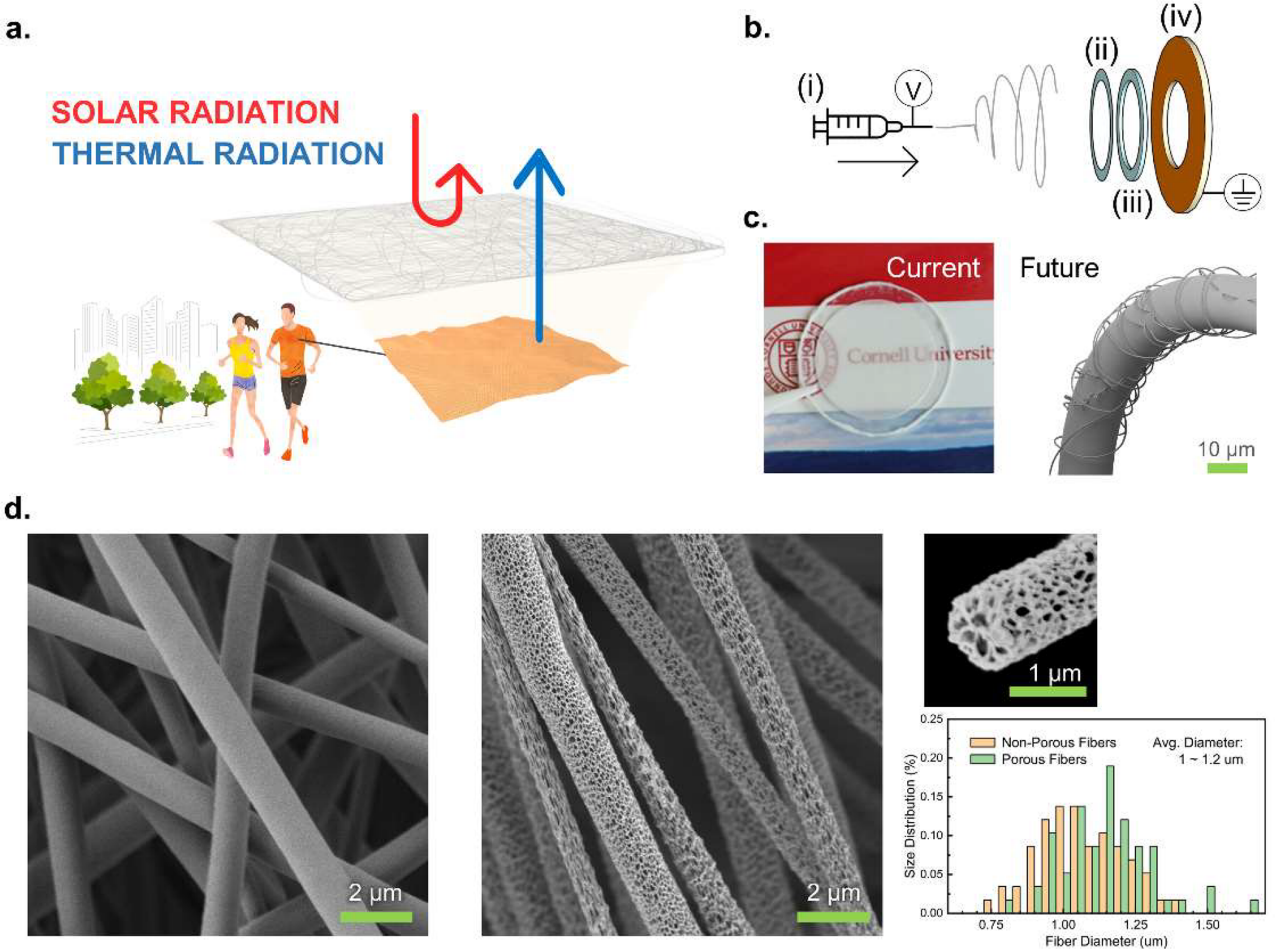
(a) Illustration of the nanoporous microfiber mat scattering solar radiation for enhanced UV protection and thermal comfort. (b) Schematic of electrospinning porous fibers with components including (i) heated solution on a syringe pump with high voltage applied on the needle, (ii) a ring-shaped Surlyn adhesive frame, (iii) a ring-shaped spacer, and (iv) a ring-shaped conductive substrate where (ii–iv) are placed in a humidity-controlled chamber. (c) Photo of an example of fiber mat and an illustration of porous fiber electrospun on conventional fiber/yarn (left to right). Logo reproduced with permission from Cornell University. (d) SEM images of nonporous fibers, porous fibers, cross-section of a porous fiber, and fiber diameter distribution plot (left to right).

Obtained fiber mats had visual variations, ranging from a completely transparent to completely white appearance, which provided qualitative estimation of both the optical transmittance and the weight per area (GSM, g/m^2^) of samples. Accurate measurements of GSM were done by stamping out every sample fiber mat from a ring-shaped frame after spectroscopic analysis, weighing only the stamped-out fiber mats using microbalance. As shown in Figure 1c, fiber mats were obtained in nonwoven form with no backing substrate. By doing so, it was possible to conduct spectroscopic analysis of only the fibers, comparing the porous against the nonporous. This comparison method also benefits the feasibility and scalability of the extremely low GSM fiber mats toward incorporating a comparable amount of material into conventional fibers as “coverspun” yarns^48,49^ (illustrated on the right of Figure 1c) and twisted nanofibers^49−51^ or further development of island-in-sea melt-spun nanofibers^25^ for adaptivity in current manufacturing systems. The parameters for electrospinning were finalized to obtain a nearly identical diameter of fibers around 1 μm for both NPF and PF samples. This is comparable to the known feature size ideal for radiative cooling material.^13,19^Figure 1d shows SEM images and the size distribution of the fibers (Figure S1). As shown in the size distribution plot, the established fabrication method consistently produced fibers with average diameter ranging between 1 and 1.2 μm. The pore size varied largely from 30 to 200 nm. Also, the pore shape was irregular, as shown in Figure 1d. The cross-sectional image shows a combination of continuous and isolated pores throughout the PF. More details on the fabrication method can be found in the Supporting Information.

The effect of the fiber diameter and introduction of nanopores is measured and analyzed by using UV–vis–NIR with an integrating sphere with respect to GSM, as shown in Figure 2a,b. While the size and shape of pores were not controlled, the optical trend proves the effect of pores when compared to nonporous fiber mat samples. In Figure 2a, solar radiation is subcategorized into UV-C (λ = 100–280 nm), UV-B (λ = 280–315 nm), UV-A (λ = 315–400 nm), visible (λ = 400–750 nm), and near-infrared (NIR, λ = 750–2500 nm) spectra to show the trend of reflectance (ρ), transmittance (τ), and absorbance (α) with respect to increase in GSM within each spectrum. Here, the experimental UV-C data ranges from 200 to 280 nm due to the limitation of the UV–vis–NIR spectrophotometer. The data points are averages calculated within the wavelengths of labeled spectrum, and average ρ of a spectrum *x*, as an example, can be written as
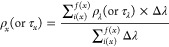
1where *ρ*_*λ*_ is the measured spectroscopic data with a set interval, Δλ. ρ and τ were based on measured data, and α was calculated from α = 1 – (ρ + τ) based on energy conservation law. Figure 2a shows that the PF mats exhibit higher reflectance and lower transmittance throughout the UV spectrum. The effect diminishes toward longer wavelength through the visible and NIR spectrum. This indicates that the nanoscale pores on the PF affect wavelengths in the near UV spectrum and shorter visible wavelength but do not interfere with longer wavelength.

**Figure 2:**
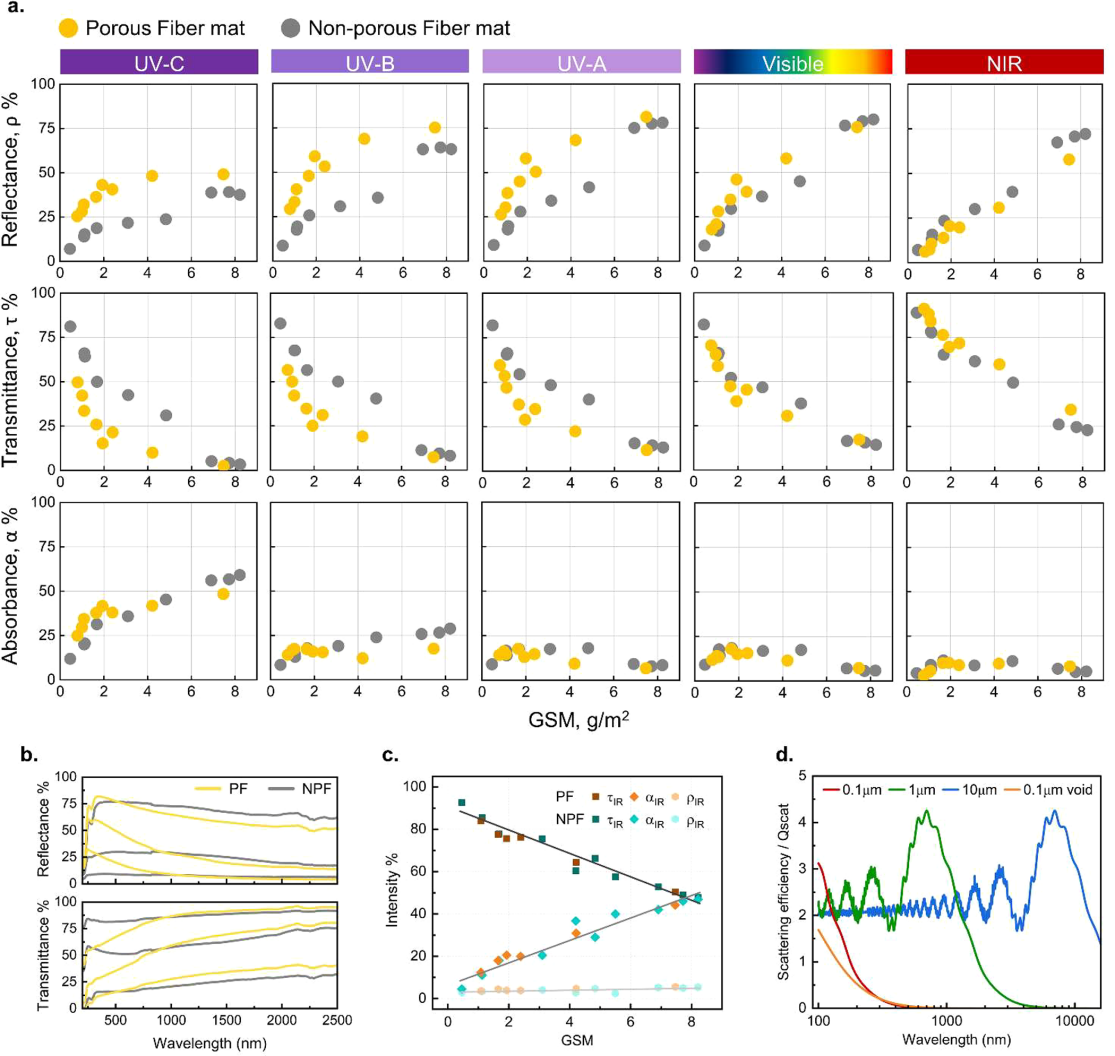
Optical properties comparison. (a) Map of spectroscopic data points of porous and nonporous fiber mats within a subcategorized solar spectrum with respect to GSM of samples. (b) Total solar reflectance and transmittance trend. (c) Spectroscopic data of porous and nonporous fiber mats within a long-wavelength infrared (LWIR) spectrum. (d) Mie scattering simulation of various sizes of particles and a pore (void) throughout the wavelengths of interest.

Figure 2b shows the *ρ*_*λ*_ and *τ*_*λ*_ of multiple samples with varying GSM from both PF and NPF represented as orange and gray, respectively. Although this is not a direct comparison of equal GSM between PF and NPF, all PFs exhibit distinct trends at shorter wavelengths, regardless of GSM. Here, this distinct trend throughout the samples proves that the previously mentioned irregular shape and size of the nanopores hold less significance. Engineering the uniformity and average diameter of the pores more precisely may shift the affected wavelengths or intensity of the optical phenomenon. With the increase in GSM, the distinction starts to diminish. This indicates that the thicker or denser the fiber mat gets, a lesser effect of nanopores will be seen. However, the NPF can only achieve an identical level of UV protection by increasing the GSM significantly, which then results in sacrificing the optical visibility, weight, and the overall cost of material. As Chen et al. demonstrated via simulation of hierarchically porous polymer film, the FT-IR spectroscopic data in the LWIR spectrum in Figure 2c agrees by showing a linear dependence of both PF and NPF on the thickness of the material, while nanoscale and microscale pores show no impact on the optical properties in the LWIR.^18^ Again, the intrinsic chemical structure of the polymer dictates the baseline absorbance in the IR regime. Therefore, utilization of naturally LWIR transparent material such as polyethylene has been pursued among radiative cooling textile research.^15,19,21^ However, the thickness of demonstrated textiles and films ranged between hundreds of microns to millimeters or was highly packed in density.^11,13,14,21^ In search of a minimum thickness or the amount of material to use, this observation highlights the benefit of nanoscale pores only to enhance the overall solar reflectance. An example of a direct LWIR spectra comparison between PF and NPF is presented in Figure S2 in Supporting Information.

A simplified Mie scattering calculation of the scattering efficiency (*Q*_scat_) is shown in Figure 2d. A shift in resonant scattering can be seen from spherical particles with three different diameters, 0.1, 1, and 10 μm, with a refractive index of 1.45 and a 0.1 μm air void with a refractive index of the medium as 1.45. Despite the simplicity in the computation of single particle cross-sectional modeling, the relationship between the feature size and affected wavelength is consistent with the experimental data of the nanoporous microfiber in this study. Both 0.1 μm particles and 0.1 μm voids represent nanoporous structures that show scattering at short wavelength. When combined, a selective optical phenomenon is achieved with UV reflective nanoscale voids and features and a highly solar reflective ∼1 μm fiber while having no impact on the ∼10 μm LWIR spectrum. Traditionally, UV protectivity has been achieved via incorporation of nanoparticles such as TiO_2_.^37,41^ Zhu et al. have achieved UV reflection with Al_2_O_3_ nanoparticles on silk fibers.^39^ A nanoporous approach is favorable in terms of a nanotoxicity and sustainability perspective.^42−44^ Preliminary experiment on utilization of the increased surface area was performed by sputtering TiO_2_ on fiber mats and showed amplified UV absorbance on PF compared to NPF (Figure S3).

Comparison of the cooling performance of PF and NPF mats is shown in Figure 3 (additional information in Supporting Information). A quartz tungsten halogen (QTH) lamp is a widely available light and produces white light, exhibiting a continuous spectrum of light from UV to IR with less intensive emission than xenon solar simulators. The tungsten filament heats to 3400 K rather than 5800 K of an ideal blackbody radiator.^52^ The thermocouples were fixed at 5 mm underneath the specimen textiles. The tested structure here is a “coated” textile rather than an incorporated structure. The presented optical phenomena and such a proof-of-concept would provide insight into optimized structures. Preferably, when knitted into an upper layer, exposed porous microscale fibers on the outer layer of the textile will scatter the solar radiation with higher efficiency as the Mie scattering theory suggests. Conduction and convection from the bottom and sides were prevented with a bulk Styrofoam base, and any reflected radiation was prevented with aluminum foil both inside and outside of the 5 mm ring-shaped spacer. There was no heating element below the skin level to replicate the skin temperature. Prepared samples here had 0.89 ± 0.1 and 1.09 ± 0.1 GSM of PF and NPF, respectively, on 159.85 ± 0.15 GSM white 100% cotton jersey knit.

**Figure 3:**
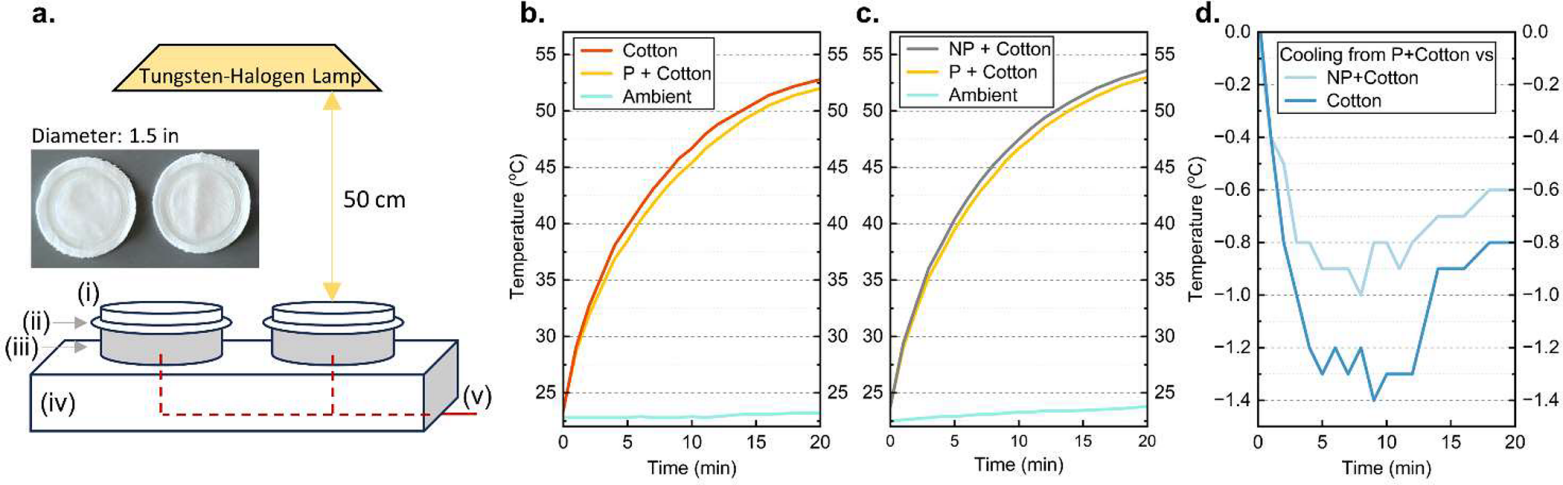
Thermal property comparison. (a) Indoor testing setup with a tungsten-halogen light source and (i) ring-shaped acrylic weights, (ii) sample fiber mats on white cotton textiles, (iii) aluminum foil wrapped ring-shaped spacers, (iv) insulated base, and (v) thermocouples. Photo with PF on cotton on left and NPF on cotton on right with a ring-shaped acrylic weight on each. (b–d) Change in skin temperature (surface temperature at 5 mm below textile) under 20 min duration of irradiance from the light source.

Figure 3b–d shows that even with a less UV intensive light source, the skin temperature (surface temperature at 5 mm below textile) of PF on cotton (P+cotton) consistently outperformed the control cotton textile and NPF on cotton (NP+cotton) by up to 1.4 and 1.0 °C, respectively. The added weight for both P+cotton and NP+cotton corresponds to <1% of cotton textile. The comparison photo in Figure 3a shows that P+cotton and NP+cotton had no visual difference. In Figure 3b, the initial temperatures for cotton, P+cotton, and ambient were 23.4, 23.5, and 22.8 °C, and the final temperatures were 52.8, 52.0, and 23.2 °C, respectively. In Figure 3c, the initial temperatures for NP+cotton, P+cotton, and ambient were 23.8, 23.8, and 22.5 °C, and the final temperatures were 53.6, 53.0, and 23.8 °C, respectively. In terms of cooling performance, both NPF and PF enhanced the solar reflectivity to achieve lower final temperatures, but PF exceeded NPF by nearly half a degree more cooling at the peak temperature (Figure 3d). Moreover, the fact that the GSM of added NPF was higher than that of PF signifies the efficiency and effectiveness. Again, the emphasis is made on using less material to achieve notable performances to address the first mentioned challenge, the cost. Mechanical durability is not discussed here because the nanoporous fibers are not meant to be used as a standalone product but to be incorporated and optimized as an additive during the extrusion, yarning, or knitting process, which would also address the second challenge of manufacturability and wearability.

Figure 4a shows a calculated transmission of solar irradiance from the measured spectroscopic data of 2 GSM samples. The transmitted energy is calculated by multiplying the obtained *τ*_*λ*_ by the standardized AM 1.5 solar spectrum (ASTM G173–03 AM1.5) as a percentage. The translated energy exhibits higher transmission from NPF, meaning about 6.68% more protection was achieved with PF. It can be noted that the effect gradually increases toward shorter wavelength. A 2 GSM PF mat blocked 70.68% of UV and 60.82% of visible radiation, while a 2 GSM NPF mat blocked 45.85% of UV and 48.08% of visible radiation. Considering that half the total solar energy is concentrated in λ = 300–710 nm as highly intensive short-wavelength radiation, prevention of UV and shorter visible radiation has a significant impact on both the health of human skin and personal thermal comfort.

**Figure 4:**
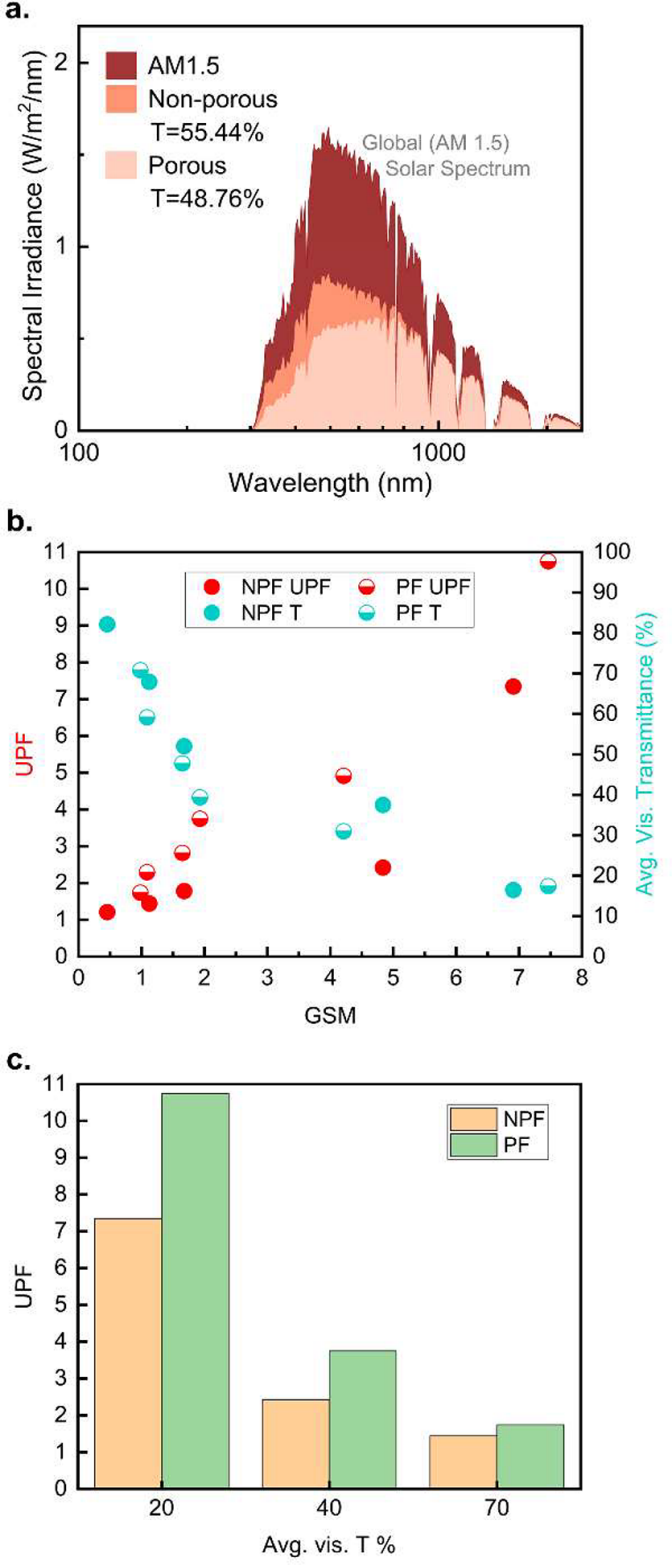
(a) Calculated total transmitted solar irradiance. (b) Calculated UV protection factor (UPF) and average τ in the visible spectrum of NPF and PF mats with respect to GSM. (c) UPF of NPF and PF mats expressed with respect to the average τ in the visible spectrum.

Figure 4b,c shows visual expressions of the UV protection factor (UPF), a standard UV protection rating method for textiles.^53^ The UPF equation is written as,
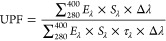
2where *E*_*λ*_ is relative erythemal spectral effectiveness, *S*_*λ*_ is solar spectral irradiance, *τ*_*λ*_ is measured transmittance, and Δλ is the measured interval. Wavelength from 280 to 400 nm covers both UV-B and UV-A to rate the UV radiation from solar radiation. Although UV-C is the most dangerous UV radiation, the standardized solar spectrum AM1.5 neglects wavelengths shorter than 300 nm, since the earth’s ozone layer and atmosphere absorb most of this radiation. Because UV-C is not considered, UV-B accounts for about 75% and UV-A about 25% of *E*_*λ*_, which describes the radiative toxicity toward human skin. A higher UPF would indicate a higher ρ or higher α of UV. With τ data used to calculate Figure 4a, the UPF values of PF and NPF were calculated to be 3.74 and 1.78, respectively. PF has a 2.1 times higher UPF than NPF. UPF is also inversely proportional to the total transmittance within the calculated spectrum, which can be translated as 73.29% of UV-B and UV-A protection with PF, whereas NPF blocks 43.86% of UV-B and UV-A. From Figure 4b, the average transmittance in the visible spectrum (avg. vis. τ) on the right *y*-axis shows an inversely proportional trend with UPF on the left *y*-axis. While the avg. vis. τ of PF and NPF shows a similar trend with respect to GSM, the UPF of PF and NPF shows a large difference in upward slope. PF mats exhibit a higher UPF rating overall. Another expression of UPF rating with respect to avg. vis. τ can be observed from Figure 4c. Here, a PF mat with ∼7.5 GSM has a ∼20% avg. vis. τ has a UPF rating of about 11. A UPF of 11 can be translated into 91% UV protection with 9% transmission by applying the inverse proportional relationship. In comparison, a 3-ply surgical mask (∼25 GSM) was measured to have a UPF of 6.7.

In summary, we report a new, minimalistic, and potentially nondisruptive method for UV protection and personal thermal comfort by reporting the optical and thermal properties of extremely thin layers of nanoporous fibers. Addition of 1 GSM of PF on an ∼160 GSM cotton t-shirt offered up to 1.4 °C cooling effect. Also, ∼7.5 GSM of the PF mat achieved a UPF rating of ∼11 which is equivalent to ∼91% UV protection. Such findings open new opportunities to further optimize with various materials and methods. Without bulky products or high-tech processes, our work signifies effectiveness with minimal amounts of material, cost, and waste. With increasing utilization of electrospinning and submicron diameter fibers in the industry,^25,32,49^ the adaptation for mass production is expected to be trouble-free.

## Supporting Information Available

Materials, fiber fabrication method, spectroscopic and thermal analysis method, additional SEM images (Figure S1), LWIR comparison (Figure S2), UV absorbance with TiO_2_ sputter deposition (Figure S3) (PDF)

## Abbreviations

UV: ultraviolet
NIR: near-infrared
LWIR: long-wavelength infrared
PF: porous fiber
NPF: nonporous fiber
GSM: grams per square meter (g/m^2^)
UPF: UV protection factor
GHG: greenhouse gas
