# Supplementary material for: Designing an Effective and Scalable UV-Protective Cooling Textile with Nanoporous Fibers
Source: Nano Lett. 2023 Nov 6;23(22):10398–405. doi: 10.1021/acs.nanolett.3c03055 (PMC10683759; doi:10.1021/acs.nanolett.3c03055)
Supplement: Supplementary file 1 — nl3c03055_si_001.pdf [file nl3c03055_si_001.pdf]

Supporting Information

Designing Effective and Scalable UV-Protective Cooling Textile with Nanoporous Fibers

Kyuin Park* and Margaret W. Frey

Department of Human Centered Design, College of Human Ecology, Cornell University, Ithaca, New York 14850.

*kp434@cornell.edu

Table of Contents

1. Experimental Methods 1

1.1. Materials 1

1.2. Fiber Fabrication Method 1-2

1.3. Spectroscopic and SEM Analysis (Figure S1) 2-3

1.4. Thermal Analysis 4

2. Figure S2. Long-wavelength infrared (LWIR) Comparison 4

3. Figure S3. UV-absorbance with TiO2 Sputter Deposition 5

1. Experimental Methods

1.1. Materials

Polylactic acid (PLA) pellets were from NatureWorks LLC (Ingeo^{TM} 6201D). Dichloromethane (DCM) was purchased from Fisher Scientific Chemicals and dimethylformamide (DMF) was from Sigma-Aldrich. A thermal-bonding transparent Surlyn® tape and a conductive copper foil tape were purchased from McMaster-Carr.

1.2. Fiber Fabrication Method

Solution of DCM with 10 wt.% of DMF was prepared. 10 wt.% of PLA pellets were added to the solution and mixed thoroughly for >3 hrs. on a shaker (Burrell Scientific). PLA solution was then transferred to a syringe with 25-gauge needle and pushed into a heated ceramic cylinder, connected to a Proportional-Integral-Derivative (PID) controller (from Omega). Two thermocouples were fixed on the inside and outside the ceramic cylinder to monitor the temperature. Non-porous fibers were spun at room temperature with the humidity of the chamber kept <40 RH%. For porous fibers, the temperature of the solution in the ceramic cylinder was kept between 38 °C to 40 °C and the humidity in the spinning chamber was kept >60 RH%. The temperature in the spinning chamber was kept at 20-22 °C. Applied voltage to the syringe needle was 15 kV and the distance from the needle to the grounded collector was 15 cm. The feed-rate of the syringe pump was controlled between 0.5 to 1.0 mL/hr. Here, the nanoscale pores were created by amplifying the effect of phase separation with three methods, electrospinning with two solvent systems, increasing humidity of the chamber, and increasing temperature of the solution.[1]

The thin acrylic sheet was laser cut in the shape of a ring with an inner diameter of 1 inch and outer diameter of 2 inch. Copper tape was cut to the same dimensions as the acrylic ring and attached to the acrylic ring. Surlyn® tape was cut to an inner diameter of 1 inch and outer diameter of 1.25 inch and lightly taped the non-adhesive side to the surface of the copper tape. The adhesive side was facing towards the needle. After

electrospinning, a free-standing textile sample was obtained by bonding another ring-shaped Surlyn® tape on top. Circular samples with clear outer frame were obtained for consistent measurements.

1.3. Spectroscopic and Scanning Electron Microscope (SEM) Analysis

The reflectance and transmittance in UV, Visible, and NIR spectrum were measured with the Agilent Cary 5000 UV-Vis-NIR Spectrophotometer with the diffuse reflectance accessory (DRA), also known as an integrating sphere. An aperture diameter of 0.75 inch was used and the beam size was consistent all throughout the sample measurements. The LWIR reflectance and transmittance were measured with the Bruker Vertex 80 FT-IR Spectrometer with a gold integrating sphere. The SEM analyses were performed using the Zeiss Gemini 500 SEM after a minimal sputter coating of gold/palladium with Denton Desk V sputter coater. Below are additional SEM images of porous and non-porous fibers. (Figure S1) SEM analysis for both NPF and PF were done by collecting extra fibers deposited on the conductive copper substrate. The average diameter of fibers was between 1 to 1.2 μm.

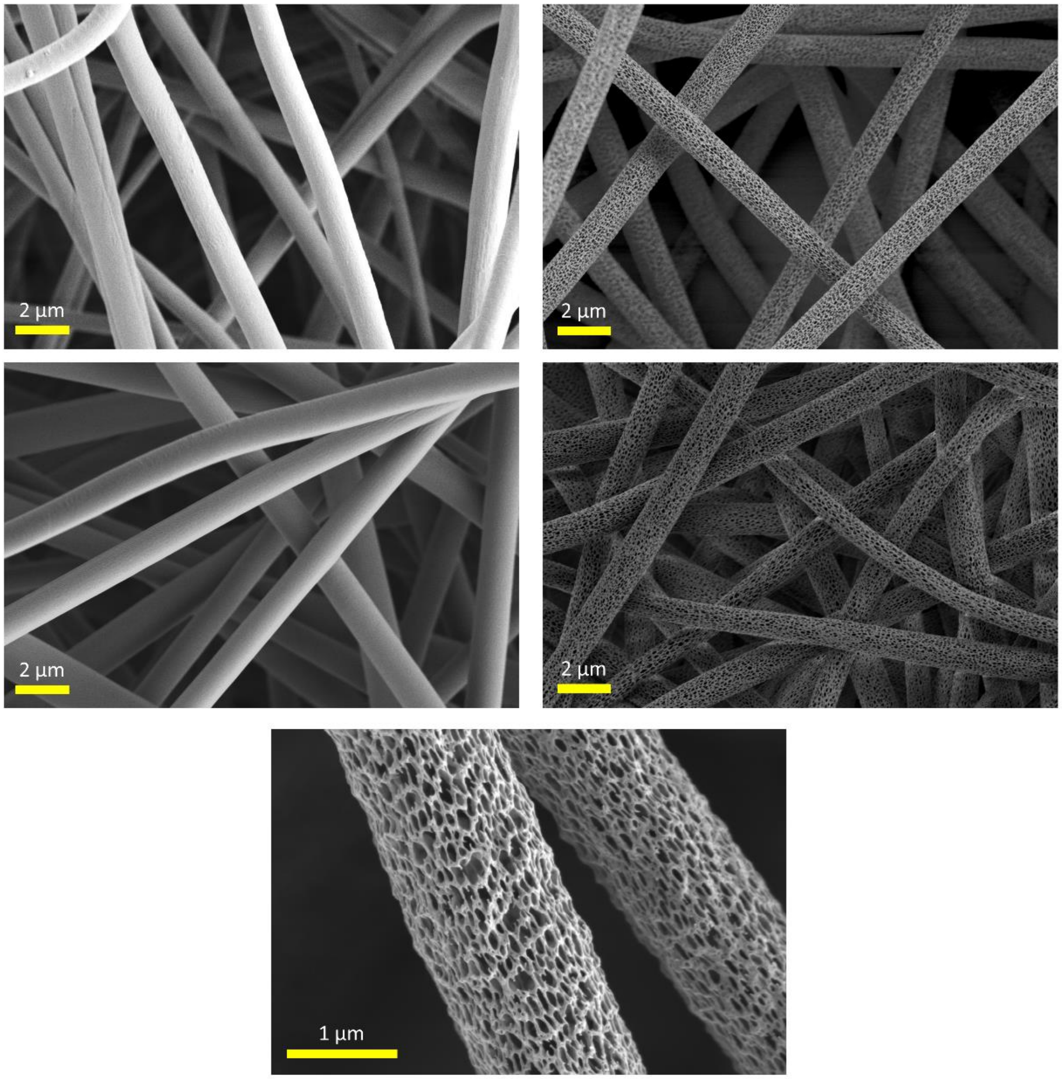

Figure S1. Additional SEM images of NPF and PF. Left two images are NPF from different samples and right two images are PF from different samples with same magnification (2 µm scale bar). Bottom higher magnification image is PF with 1 µm scale bar.

1.4. Thermal Analysis

Comparison between cotton, PF with cotton, and NPF with cotton were conducted with two separate measurements. The 600 W quartz tungsten halogen (QTH) lamp (from HDX) was placed 50 cm directly above the setup. Two thermocouples were connected to Amprobe TMD-50 thermometer (accuracy of 0.1%). PF and NPF were transferred to cotton by lightly compressing each fiber mat on two separate cotton textiles at the center with a 1-inch diameter cylinder block and then lifting ring-shaped Surlyn frame, leaving only the fiber mat directly deposited on cotton. Three cotton textiles were cut with a 1.5-inch diameter fabric cutter from a larger fabric and weighed.

2. Long-wavelength infrared (LWIR) Comparison

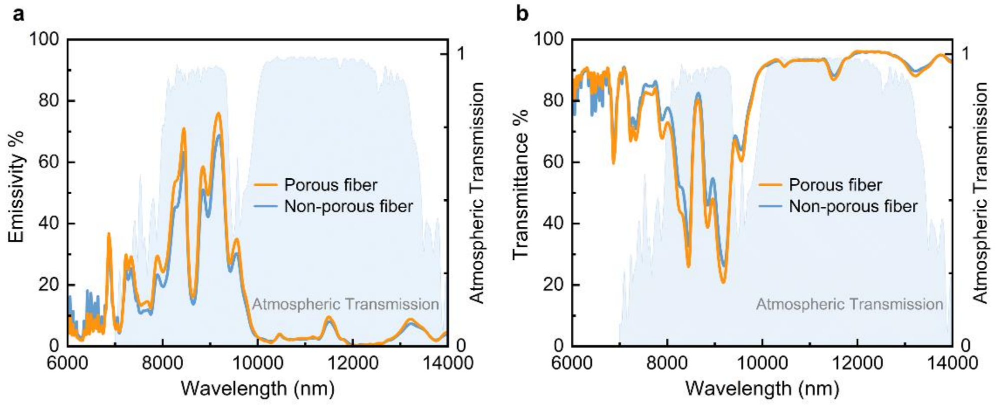

Figure S2. Direct comparison of LWIR emissivity and transmittance of 2 GSM PF and NPF samples.

FT-IR spectra of polylactic acid (PLA) fiber mats show a typical absorption of PLA. Bending vibrations of -CH_3 at ~6.8-7.3 µm and -C-O stretching between ~7.7-10.0 µm can be observed. Although not shown in above figure, a sharp absorption peak around 5.7 µm can be observed.

3. UV-Absorbance with TiO_2 Sputter Deposition

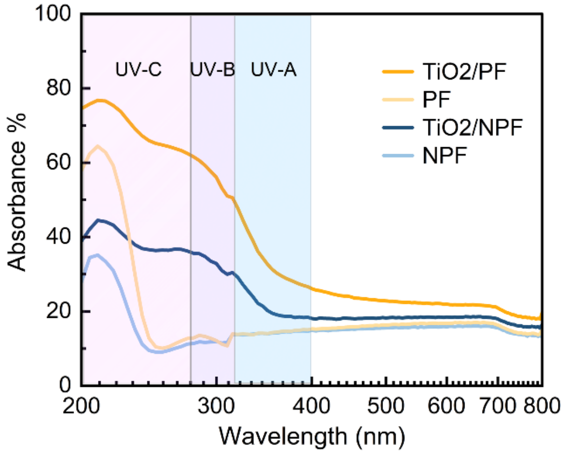

Figure S3. Enhanced UV-absorbance with TiO_2 thin film sputter deposition.

With the 3-Gun Sputter System at Cornell Center for Materials Research (CCMR), TiO_2 was sputtered on PF mat and NPF mat, with nearly identical GSM, in the same batch. The deposition rate was ~1 Å/s of total ~10 nm thickness. Introduction of nanoscale pores expanded the total surface area of the fiber and showed amplification of UV-absorbance property when compared to non-porous fiber sample.

Reference

(1) Li, Y.; Lim, C. T.; Kotaki, M. Study on Structural and Mechanical Properties of Porous PLA Nanofibers Electrospun by Channel-Based Electrospinning System. *Polymer* **2015**, *56*, 572–580. https://doi.org/10.1016/j.polymer.2014.10.073.
